# Nocardia rubra cell-wall skeleton mitigates whole abdominal irradiation-induced intestinal injury via regulating macrophage function

**DOI:** 10.1093/burnst/tkad045

**Published:** 2024-03-04

**Authors:** Lingling Wu, Long Chen, Huijuan Li, Yawei Wang, Kexin Xu, Wanchao Chen, Aihua Zhang, Yu Wang, Chunmeng Shi

**Affiliations:** Department of Toxicology, School of Public Health, Guizhou Medical University, Guiyang, 550025, China; State Key Laboratory of Trauma and Chemical Poisoning, Third Military Medical University (Army Medical University), 400038, Chongqing, China; State Key Laboratory of Trauma and Chemical Poisoning, Third Military Medical University (Army Medical University), 400038, Chongqing, China; State Key Laboratory of Trauma and Chemical Poisoning, Third Military Medical University (Army Medical University), 400038, Chongqing, China; State Key Laboratory of Trauma and Chemical Poisoning, Third Military Medical University (Army Medical University), 400038, Chongqing, China; State Key Laboratory of Trauma and Chemical Poisoning, Third Military Medical University (Army Medical University), 400038, Chongqing, China; College of Biological Engineering, Chongqing University 400044, Chongqing, China; State Key Laboratory of Trauma and Chemical Poisoning, Third Military Medical University (Army Medical University), 400038, Chongqing, China; Department of Toxicology, School of Public Health, Guizhou Medical University, Guiyang, 550025, China; State Key Laboratory of Trauma and Chemical Poisoning, Third Military Medical University (Army Medical University), 400038, Chongqing, China; Department of Toxicology, School of Public Health, Guizhou Medical University, Guiyang, 550025, China; State Key Laboratory of Trauma and Chemical Poisoning, Third Military Medical University (Army Medical University), 400038, Chongqing, China

**Keywords:** Nocardia rubra cell wall skeleton, Ionizing radiation, Radiation-induced intestinal injury, Intestinal flora, Radiation protection, Peritoneal macrophages

## Abstract

**Background:**

Ionizing radiation (IR)-induced intestinal injury is a major side effect and dose-limiting toxicity in patients receiving radiotherapy. There is an urgent need to identify an effective and safe radioprotectant to reduce radiation-induced intestinal injury. Immunoregulation is considered an effective strategy against IR-induced injury. The purpose of this article was to investigate the protective effect of Nocardia rubra cell wall skeleton (Nr-CWS), an immunomodulator, on radiation-induced intestinal damage and to explore its potential mechanism.

**Methods:**

C57BL/6 J male mice exposed to 12 Gy whole abdominal irradiation (WAI) were examined for survival rate, morphology and function of the intestine and spleen, as well as the gut microbiota, to comprehensively evaluate the therapeutic effects of Nr-CWS on radiation-induced intestinal and splenetic injury. To further elucidate the underlying mechanisms of Nr-CWS-mediated intestinal protection, macrophages were depleted by clodronate liposomes to determine whether Nr-CWS-induced radioprotection is macrophage dependent, and the function of peritoneal macrophages stimulated by Nr-CWS was detected *in vitro*.

**Results:**

Our data showed that Nr-CWS promoted the recovery of intestinal barrier function, enhanced leucine-rich repeat-containing G protein-coupled receptor 5^+^ intestinal stem cell survival and the regeneration of intestinal epithelial cells, maintained intestinal flora homeostasis, protected spleen morphology and function, and improved the outcome of mice exposed to 12 Gy WAI. Mechanistic studies indicated that Nr-CWS recruited macrophages to reduce WAI-induced intestinal damage. Moreover, macrophage depletion by clodronate liposomes blocked Nr-CWS-induced radioprotection. *In vitro*, we found that Nr-CWS activated the nuclear factor kappa-B signaling pathway and promoted the phagocytosis and migration ability of peritoneal macrophages.

**Conclusions:**

Our study suggests the therapeutic effect of Nr-CWS on radiation-induced intestinal injury, and provides possible therapeutic strategy and potential preventive and therapeutic drugs to alleviate it.

Highlights

- Nocardia rubra cell wall skeleton, as an approved National Category II New Drug, was proven to possess beneficial properties against radiation-induced intestinal injury.
- Nocardia rubra cell wall skeleton can effectively ameliorate WAI-induced intestinal barrier structural and functional injury and promote the regeneration of the intestinal epithelium.
- Nocardia rubra cell wall skeleton can improve intestinal bacterial flora homeostasis and alter the composition of the gut microbiota in WAI mice.
- Nocardia rubra cell wall skeleton protects the morphology and function of the spleen against radiation-induced spleen damage.
- Nocardia rubra cell wall skeleton-induced radioprotection is macrophage dependent. Nr-CWS activated the NF-κB signaling pathway and promoted the phagocytosis and migration of macrophages.

## Background

Ionizing radiation (IR) has been increasingly used to treat multiple malignant tumors [1]. Radiotherapy planning and delivery methods have improved substantially, but toxicity to normal ‘healthy’ tissue is still a problem [2,3]. High doses of IR may cause fatal acute radiation syndromes and dose-dependent damage to the hematopoietic, gastrointestinal, skin, cardiovascular or nervous systems [4,5]. In particular, rapidly proliferating cells of the gastrointestinal and hematopoietic systems are especially susceptible to radiation effects [6,7]. In the radiotherapy of abdominal or pelvic solid tumors, the small intestine is a susceptible and common site of radiation-induced injury. Intestinal damage induced by radiotherapy can cause gastrointestinal dysfunction, such as diarrhea, nausea, vomiting, bleeding, bacteremia, perforation and even death [5,8,9]. During radiation therapy for patients with malignant tumors, both gastrointestinal and hematopoietic systems are at risk of serious complications, which seriously affect quality of life. In contrast to hematopoietic damage, which can be rescued by bone marrow transplantation, there is almost no effective therapeutic intestinal radioprotectant in routine clinical use [10]. Therefore, developing effective therapies and drugs to prevent or treat irradiation-induced gastrointestinal toxicity is urgently needed.

Radiation-induced acute tissue toxicity is associated with the inflammatory and immune responses of the tissue [11]. A previous study on radiation and radiation-combined injury indicated that exposure to IR resulted in dose-dependent losses of immune cell subsets. High-dose radiation exposure can cause immune decline or immunosuppression [12]. Substantial evidence has demonstrated the therapeutic benefits of immunoregulation against IR-induced injury. Intestinal macrophages are one of the largest groups of macrophages in the body, and they are also the main participants in the establishment and maintenance of intestinal environmental balance [13–16]. Macrophages are a new therapeutic target in radiation injury [17]. Yi *et al*. showed that epigallocatechin gallate could protect against high-dose IR-induced injury by enhancing macrophage phagocytosis [18]. In addition, some radiation protective agents (Gmur003M, toll-like receptor 9 agonists, polyphenol acetate 7-diacetoxy-4-methylthiocoumarin, etc.) are partly based on triggering macrophages to repair radiation-damaged intestinal epithelium [11,19,20]. Macrophages, as a regulatory target in radiation protection, deserve further investigation.

The Nocardia rubra cell wall skeleton (Nr-CWS), an innate immunomodulatory and antitumor agent [14,21], was first reported in 1976 by Azuma *et al*. [22]. As an approved National Category II New Drug, Nr-CWS has been widely used in human cervical diseases and cancers [23–25]. An increasing number of studies have demonstrated that Nr-CWS can activate macrophages [21,26], natural killer cells [27] and T cells [28,29], which suggests its potential effect on immunoregulation. However, the role of Nr-CWS in ionizing radiation-induced intestinal injury is still unclear. We speculated that it might have a potential anti-radiation effect, considering its immunoregulatory function. Therefore, we aimed to assess the effect of Nr-CWS on alleviating IR-induced intestinal injury, and to further investigate the underlying regulatory mechanisms in mice.

In this study, we sought to investigate whether Nr-CWS might ameliorate radiation-induced acute intestinal injury using whole abdominal irradiation (WAI) mouse models. We found that Nr–CWS improved intestinal and spleen morphology and function, and maintained intestinal flora homeostasis, which increased the survival rate of irradiated mice. Nr-CWS-induced radioprotection was macrophage dependent, activated the nuclear factor kappa-B (NF-κB) signaling pathway and promoted macrophage phagocytosis and migration. Thus, our findings demonstrate the protective and therapeutic potential of Nr-CWS in alleviating WAI-induced intestinal injury in a preclinical setting.

## Methods

### Animals and ethical approval

Male C57BL/6 J mice (8–10 weeks old) were purchased from the Laboratory Animal Center of the Army Medical University. All mice were housed in a temperature- and humidity-controlled room with a 12 h light/dark cycle and provided *ad libitum* access to food and water. The adult mice were kept in quarantine for 1 week before the commencement of the animal models and assignment to groups. In this study, all experiments involving animals were approved by the Ethics committee and performed in accordance with the Animal Care and Use Committee Guidelines of the Army Military Medical University.

### WAI and Nr-CWS treatment

**Figure 1:**
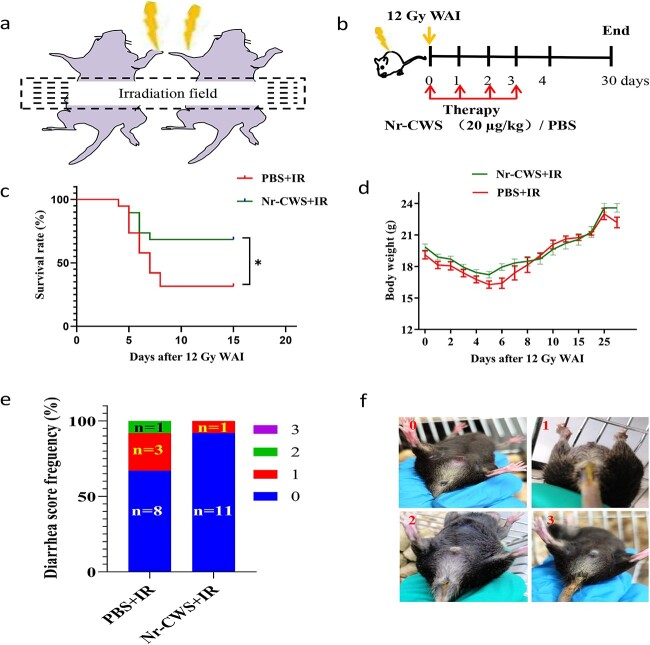
Nr-CWS increases survival of mice after WAI. As shown in (**a**) the area outside the radiation was shielded with a lead plate to expose only the whole abdomen to induce acute intestinal injury. Mice immediately received intraperitoneal administration of Nr-CWS (20 μg/kg) once a day for 3 days after 12 Gy WAI. PBS + IR group mice received the same volume and frequency of sterile PBS as the treated mice at illustrated in (**b**). (**c**) Kaplan–Meier survival analysis of mice treated with Nr-CWS/PBS after exposure to 12 Gy WAI, for Nr-CWS + IR group *vs* PBS + IR group, by log-rank test (^*^p < 0.05); n = 22. (**d**) Body weight of irradiated mice and mice treated with Nr-CWS or PBS after 12 Gy WAI; n = 22. (**e**) Diarrhea score of Nr-CWS/PBS-treated mice on the third day after 12 Gy WAI. (**f**) Diarrhea standard schematic diagram (the numbers indicate diarrhea scores, from 0 to 3 points. 0: refers to normal stool; 1: refers to mild wet stool; 2: refers to unformed stool; 3: refers to watery stool); n = 12. *Nr-CWS* nocardia rubra cell wall skeleton, *WAI* whole abdominal irradiation, *PBS* phosphate-buffered saline, *IR* ionizing radiation

An X-Ray irradiator (160–225 instrument, Branford) at a dose rate of 0.9 Gy/min was used for all experimental mice under anesthesia. All male mice were exposed to a single dose of 12 Gy in a specific steel chamber; the radiation dose was monitored by a dose rate meter. The entire abdomen of each mouse was exposed to radiation while the other parts of the mouse were shielded with lead shielding (Figure 1a). Post-irradiation, the mice were returned to the animal facility for daily treatment and observation. In the survival experiments (n = 22 per group), irradiated-mice were randomized into phosphate-buffered saline (PBS) + IR group and Nr-CWS + IR group. In the remaining experiments, mice were randomized into PBS + IR and Nr-CWS + IR groups that received 12 Gy WAI and a NO IR group that were sham-irradiated (n = 12 per group). Nr-CWS (20 μg/kg) was administered immediately after WAI. The relevant figure legends provide the doses and schedules for administering Nr-CWS and radiation.

### Sample collection and organ index determination

Stool samples were collected from the colon under aseptic conditions and stored at −80°C immediately for further use. After the mice were euthanized, the spleen was collected, immediately weighed and then fixed in 4% formaldehyde. Spleen indices were expressed relative to the body weight of mice. In addition, the intestines of mice were dissected after euthanasia, washed in cold PBS to prevent contamination and stored at −80°C for later use and fixed in 4% paraformaldehyde. All tissue samples were taken from the same location in each animal.

### Histopathological studies

The small intestine and spleen were fixed in 4% neutral-buffered formalin for 24 h. Next, tissues were dehydrated using gradient alcohol before paraffin embedding and sliced (cut into 4 μm slices) with a slicing machine. Hematoxylin and eosin (H&E) staining and Aligen blue Periodic-acid-Schiff (AB-PAS), as previously described [30], were observed and photographed with a fluorescence microscope (LAS AF Lite).

#### Determination of surviving crypts

The number of surviving crypts per circumference of transverse intestinal section was determined from H&E-stained sections based on prior research. Surviving crypts contain five or more adjacent chromophilic non-Paneth cells, at least one Paneth cell and a lumen.

#### Determination of villus length and crypt depth

Villus length and crypt depth were independently and objectively quantified and analyzed by photographing the H&E-stained sections using a LAS AF Lite or VS2000. The villus length was determined from the crypt–villus junction to the tip of the villus. For each group, at least 50–80 villi were measured from different locations in the jejunum. Moreover, the crypt depth quantified the height from the bottom of the crypt to the crypt–villus junction.

#### Extramedullary hematopoiesis score

The spleen scoring system was based on the amount and patterns of extramedullary hematopoiesis (EMH) [31]. Supporting information is provided in the appendix.

### Immunohistochemistry and immunofluorescence analysis

At 4 h and 3.5 d after WAI, the intestine and spleen were collected and fixed in 4% paraformaldehyde for 24 h. Next, tissues were dehydrated, embedded, sectioned, affixed to detachable glass slides, baked in an oven, dewaxed to water and boiled with Tris/EDTA (pH 9.0) or citric acid (pH 6.5) at 98°C for 20 min until after antigen repair. For immunohistochemistry, sections were soaked in 3% H_2_O_2_ for 15 min to remove tissue contents. Furthermore, sections were blocked with goat serum for 40 min at room temperature and then incubated with primary antibodies at 4°C overnight, including proliferating cell nuclear antigen (PCNA) antibody (CST, 1 : 200) and leucine-rich repeat-containing G protein-coupled receptor 5 (Lgr5) antibody (ProteinTech, 1 : 200). Then, sections were incubated with the appropriate secondary antibody for 1 h. Next, diluted diaminobenzidine was used to detect positive cells. For immunofluorescence (IF), deparaffinized sections were rehydrated, and antigens were retrieved using Tris-/EDTA (pH 9.0) or citric acid (pH 6.5). Next, sections were blocked with goat serum for 40 min at room temperature and then incubated with primary antibodies at 4°C overnight, including phosphorylated histone 2Ax (γH2AX) antibody (CST, 1 : 200), zonula occludens-1 (ZO-1) antibody (ProteinTech,1 : 1000), lysozyme antibody (Abcam, 1 : 1000), cleaved capase3 antibody (CST, 1 : 200), F4/80 antibody (Abcam, 1 : 200) and ki-67 antibody (Abcam, 1 : 200). The next day, all sections were incubated with the appropriate fluorescent secondary antibody (1 : 1000) for 1 h, the nuclei were stained with 4,6-diamidino-2-phenylindole (DAPI) and images were captured using a fluorescence microscope under a dark environment (Olympus BX51).

### TdT-mediated dUTP nick-end labeling assay

Apoptotic cells in tissues were detected *in situ* using TdT-mediated dUTP nick-end labeling (TUNEL) staining. Mice were sacrificed 6 h post-WAI, and small intestine and spleen tissue were harvested and fixed in 4% formaldehyde. The 4 μm-thick sections were treated according to the manufacturer’s protocols (Roche, USA).

### Bacterial diversity analysis

The total genomic DNA was extracted from the fecal samples using the Cetyltrimethylammonium Bromide/sodium dodecyl sulfate (CTAB/SDS) method, and the purity and concentration of DNA were monitored on 1% agarose gels. According to the concentration, DNA was diluted to 1 ng/μl using sterile water. The V3–V4 regions of 16S rDNA genes were amplified by specific primers, 341F: CCTAYGGGRBGCASCA and 806R: GGACTACNNGGGTATCTAAT with barcode sequences. Thermal cycling consisted of initial denaturation at 98°C for 1 min, followed by 30 cycles of denaturation at 98°C for 10 s, annealing at 50°C for 30 s and elongation at 72°C for 30 s, and finally, 72°C for 5 min. Sequencing libraries were generated using a TruSeq® DNA PCR-Free Sample Preparation Kit (Illumina) following the manufacturer’s recommendations, and index codes were added. The library quality was assessed on the Qubit@ 2.0 Fluorometer (Thermo Scientific) and Agilent Bioanalyzer 2100 system. Finally, the library was sequenced on an Illumina NovaSeq 6000 platform and 250 bp paired-end reads were generated.

The raw microbiome data obtained from sequencing were a paired-end sequence saved in ‘fastq’ and then assembled by FLASH software. The sequences with ≥97% similarity were assigned to the same operational taxonomic unit (OTU) through UPARSE software. The representative sequences (Silva 132 for 16S, UNITE for ITS) for each OTU were screened for further annotation. Moreover, the Silva database (Version 132) was used based on the RDP classifier. All microbiota data determined by 16S DNA are provided in supplementary Table 1, see online supplementary material.

### Quantitative real-time PCR

Total RNA was extracted using TRIzol agent (Invitrogen) according to the manufacturer’s protocol. cDNA synthesis was performed following the manufacturer’s protocol with a RevertAid First Strand cDNA Synthesis Kit (Thermo Fisher Scientific). Quantitative real-time PCR (RT-PCR) was performed using SYBR Green qPCR master mix (Takara) according to the manufacturer’s protocol. The primer sequences for RT-PCR are listed in supplementary Table 2, see online supplementary material.

### Macrophage depletion in mice

Macrophages in mice were depleted using clodronate liposomes (Liposoma, CP-005-005). The suspensions of control PBS liposomes and clodronate liposomes were delivered intraperitoneally (i.p.) (200 μl) to deplete macrophages 72 h before exposure to 12 Gy WAI. Macrophage depletion was subjected to flow cytometry 72 h after treatment, and the expression of APC-F4/80 (BioLegend, #123113) was analyzed.

### Peritoneal macrophage preparation

C57BL/6 J mice (6–10 weeks) were treated i.p. with 1 ml of 3% thioglycolic acid medium per day to elicit macrophages for 3 d. Three days later, the mice were sacrificed and injected i.p. with 5 ml of cold PBS. Then macrophages were collected and cultured in DMEM containing 10% fetal bovine serum and 1% streptomycin/penicillin. After incubation at 37°C/5% CO_2_ for 2 h, the floating cells were removed by rinsing with PBS, and the remaining adherent cells were regarded as peritoneal macrophages (PMs).

### Measurement of phagocytosis

PMs were treated with 0.1 μg/ml Nr-CWS for 24 h, incubated with fluorescent latex microbeads (Sigma, L3030) for 1.5 h, and then measured by microscopy or flow cytometry. For microscopic analysis, the uptake of latex microbeads into PMs was captured by microscopy and quantified by estimating the percentage of macrophages containing one latex microbead. For flow cytometry analysis, the percentage of phagocytosed latex microbeads was assessed to determine the effect of Nr-CWS on macrophage phagocytosis.

### Measurement of migration

A total of 15,000 PMs were seeded into 24-well cell plates with 8 mm pores (Corning, #3422) and treated with 0.1 μg/ml Nr-CWS for 24 h. The cells on the upper surface of the filters were removed with a cotton swab. For visualization, cells on lower filter surfaces were fixed with paraformaldehyde and stained with DAPI. Then, the cells were imaged by microscopy and quantified by estimating three to five fields per filter. Data are presented as migrated cells per field.

### Western blotting

PMs were lysed in RIPA buffer containing protein inhibitor and phosphatase inhibitor (Roche) on ice for 30 min and then centrifuged at 15,000 × g for 15 min at 4°C to obtain total protein lysates. Protein concentrations were determined using a Bradford protein concentration assay kit (Beyotime). The same amounts of proteins were subjected to western blot analysis by Sodium dodecyl sulfate polycarylamide gel electrophoresis (SDS–PAGE). The primary antibodies used were anti-β-actin (Abcam, 1 : 1000), anti-P65 (CST, 1 : 1000) and anti-P-P65 (CST, 1 : 1000). Bound antibodies were detected with Horse radish peroxidase (HRP)-conjugated secondary antibodies (Beyotime, 1 : 2000) at room temperature for 1 h and by an ECL kit (Thermo Scientific, Waltham, USA). β-Actin was used as the loading control for whole cell lysates.

### Statistical analysis

The experimental groupings were performed in a balanced, blinded and randomized manner. Kaplan–Meier statistics were used to analyze the survival rate of mice. All data are expressed as the mean ± standard deviation. All data analyses were performed using GraphPad Prism 8.0 software. The significance between two groups was evaluated by using Student’s t test. The comparison of three or multiple groups was analyzed via one-way Analysis of variance (ANOVA), followed by Fisher’s least significant difference tests. The 16S rDNA sequencing data were analyzed with R software (Version 4.0.6) or UPARSE software. OTU used the RDP classifier to annotate taxonomic information for each representative sequence (Silva132 for 16S, UNITE for ITS). Nonmetric multidimensional scaling (NMDS) analyses based on the Bray–Curtis dissimilarity distance matrices and phyloseq packages were performed to compare the heterogeneous community structure of the gut microbiota of different groups. Cluster analysis was carried out by clustering diagrams, Euclidean distance and hierarchical clustering. Linear discriminant analysis (LDA) effect size (LEfSe) was utilized to confirm differences in the abundances of individual taxa between the two groups. LEfSe was used for the quantitative analysis of biomarkers within different groups (LDA score > 2, *p* < 0.05). The statistical significance threshold was set at 0.05, and in all cases *p* < 0.05 was considered statistically significant. In addition, asterisks denote statistical significance (ns, nonsignificant; ^*^p < 0.05, ^*^^*^p < 0.01, ^*^^*^^*^p < 0.001).

## Results

### Nr-CWS increases survival in mice after WAI

WAI caused acute intestinal injuries while protecting other tissues and organs, such as the bone marrow (Figure 1a). First, mice were exposed to 12 Gy WAI and treated with Nr-CWS immediately until the third day post-radiation (Figure 1b). Interestingly, the survival rate of Nr-CWS-treated mice was 70%, while that of PBS-treated mice was only 30% in the radiation model (Figure 1c). Moreover, Nr-CWS treatment relieved the trend of weight loss in the irradiated mice (Figure 1d). Furthermore, we observed the daily diarrhea of mice exposed to 12 Gy WAI and found that Nr-CWS treatment significantly reduced the occurrence of diarrhea at the third day post-radiation (Figure 1e, f). These results indicated that Nr-CWS treatment significantly protected against WAI-induced lethality in mice.

### Nr-CWS alleviates WAI-induced intestinal injury in mice

**Figure 2:**
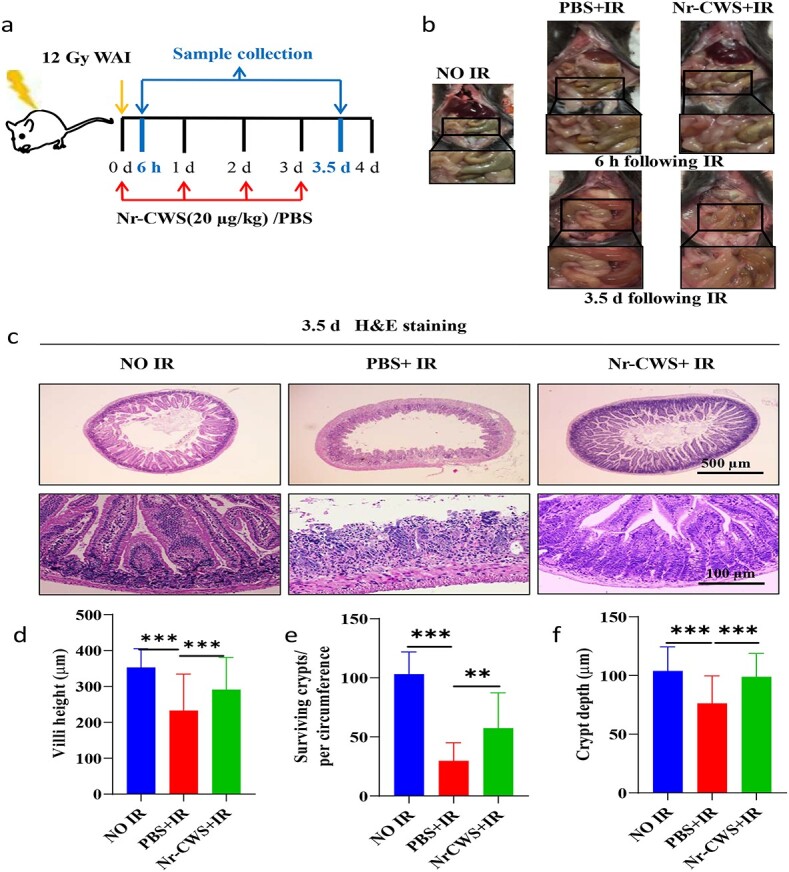
Nr-CWS protects the intestinal morphology of mice after 12 Gy WAI. Mice were treated with Nr-CWS intraperitoneal injection immediately after 12 Gy WAI. Mice in the PBS + IR group received the same frequency and volume of PBS. Intestine and other specimens were collected from Nr-CWS/PBS/and un-irradiated mice at 6 h and 3.5 d after 12 Gy WAI, as illustrated in (**a**). (**b**) Representative images of the anatomical intestinal tracts. (**c**) H&E staining sections of intestine; scale bars: 500 μm (top) and 100 μm (bottom). (**d**) Villus height at 3.5 d after 12 Gy WAI. (**e**) Average number of surviving crypts per circumference at 3.5 d after 12 Gy WAI. (**f**) Average crypt depth per section at 3.5 d after WAI; n = 8 (^**^p < 0.01, ^***^p < 0.001). *Nr-CWS* nocardia rubra cell wall skeleton, *WAI* whole abdominal irradiation, *PBS* phosphate-buffered saline, *IR* ionizing radiation, *H&E* hematoxylin and eosin

To further examine the effects of Nr-CWS on radiation-induced intestinal injuries in irradiated mice, we evaluated the injury of the small intestines following 12 Gy WAI with Nr-CWS or PBS treatment at 6 h and 3.5 d following radiation exposure. As shown in Figure 2b, WAI caused the contents of the small intestine to slim down and blood or watery diarrhea appeared in mice exposed to WAI at 3.5 d. In contrast, X-ray treatment reduced the degree of injury and maintained the contents of the intestinal tract near the normal state. Visually, these phenomena were supported via H&E staining of the intestine, demonstrating the loss of crypts and the reduction in villus height post-radiation. In contrast, the crypt–villus architecture and surviving crypt number of the small intestinal tracts in irradiated mice treated with Nr-CWS were well-preserved (Figure 2c–f).

**Figure 3:**
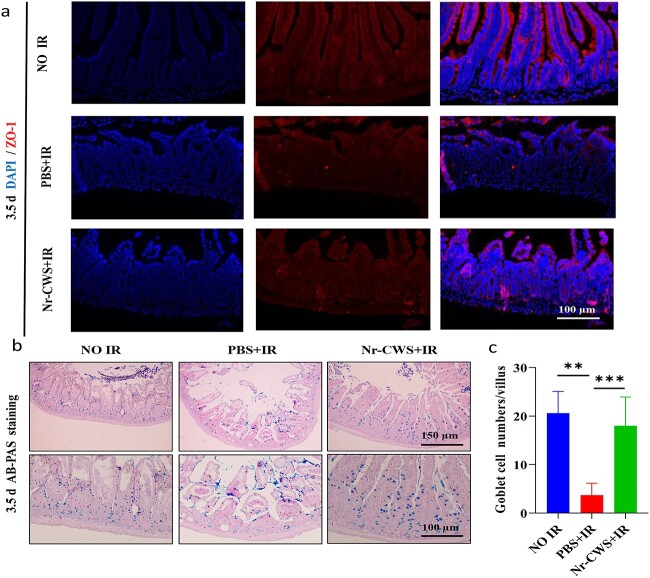
Nr-CWS treatment recovers the intestinal barrier function of WAI-induced intestinal injury in mice. (**a**) Representative immunofluorescence staining of ZO-1 in the intestinal sections from Nr-CWS- or PBS-treated mice at 3.5 d post-irradiation; n = 3. Scale bar: 100 μm. (**b**) Representative images of Alligan–blue Periodic Acid Schiff (AB-PAS) staining after 12 Gy WAI, n = 8; scale bars: 150 μm (top) and 100 μm (bottom). (**c**) Quantification of goblet cells based on AB-PAS staining, the average number of goblet cells per villus; n = 8 (^**^p < 0.01, ^***^p < 0.001). *Nr-CWS* nocardia rubra cell wall skeleton. *PBS* phosphate buffered saline, *IR* ionizing radiation, *ZO-1* zonula occludins-1, *AB-PAS* Alligan–blue Periodic Acid Schiff

The small intestine villi provide the appearance of passivation and shedding post-radiation, which means that the secretion of digestive enzymes was reduced and the intestinal absorption or barrier function might be damaged [32,33]. Indeed IF staining of the tight junction protein ZO-1 revealed significantly higher ZO-1 expression in the Nr-CWS + IR group than in the PBS + IR group at 3.5 d post-radiation. These results suggested that Nr-CWS treatment alleviated radiation-induced intestinal epithelial cell injury and induced a functional recovery of intestinal epithelial integrity (Figure 3a). Goblet cells (indicated by secreted mucin granules) play a significant role in barrier function, which can lubricate epithelial surfaces and protect epithelial cells [34]. The quantification of AB-PAS staining showed that the number of goblet cells was markedly decreased in mice at 3.5 d after WAI, however, administration of Nr-CWS distinctly increased the number of goblet cells in irradiated mice (Figure 3b, c). These data indicated that Nr-CWS might have a beneficial effect on radiation-induced intestinal injury.

### Nr-CWS ameliorates DNA damage and cell apoptosis in the intestinal crypts of WAI-exposed mice

**Figure 4:**
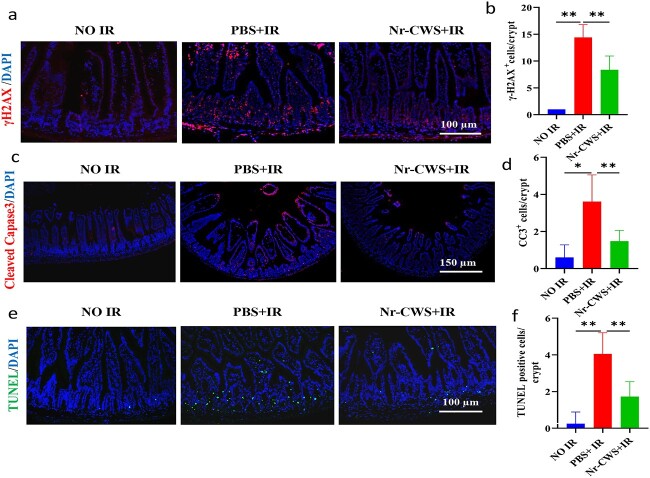
Nr-CWS ameliorates DNA damage and cell apoptosis in the intestinal crypts of WAI-exposed mice. (**a**) Representative immunofluorescence images of γH2AX in the intestine of mice (red, γH2AX; blue, DAPI); scale bar: 100 μm. (**b**) Quantitation of γH2AX per crypt. (**c**, **e**) Apoptosis in the intestinal crypt cells was evaluated by CC3 immunofluorescence and TUNEL assay, respectively; scale bar: 150 μm and 100 μm. (**d**, **f**) Quantitation of CC3- and TUNEL-positive cells per crypt; n = 8. (^*^p < 0.05, ^**^p < 0.01). *Nr-CWS* nocardia rubra cell wall skeleton, *PBS* phosphate-buffered saline, *IR* ionizing radiation, *CC3* cleaved capase3, *γH2Ax* phosphorylated histone 2Ax, *TUNEL* TdT-mediated dUTP nick-end labeling, *DAPI* 4,6-diamidino-2-phenylindole

Previous studies have shown that radiation exposure causes DNA damage, which causes apoptosis [35]. Phosphorylation of histone H2AX is a marker of DNA damage, especially when damage involves the induction of DNA double-strand breaks (DSBs) [36]. IF staining at 6 h after irradiation was used to observe whether Nr-CWS improved the genetic stability of the small intestine of irradiated mice. The results showed that the expression of γH2AX cells in the Nr-CWS + IR group was lower than that in the PBS + IR group (Figure 4a, b). Caspases are a vital gene family that maintains homeostasis by regulating apoptosis and inflammation [35]. Therefore, to further determine the effects on cell apoptosis, we performed cleaved capase3 staining (Figure 4c, d) and TUNEL staining (Figure 4e. f) of the intestinal sections at 6 h after WAI. These data showed that the PBS + IR group had more apoptotic cells than the Nr-CWS + IR group. These results suggested that Nr-CWS improved DNA damage and cell apoptosis in the intestinal crypts of WAI-exposed mice.

### Nr-CWS enhances Lgr5^+^ intestinal stem cell survival and maintains the regeneration of intestinal epithelial cells in irradiated mice

**Figure 5:**
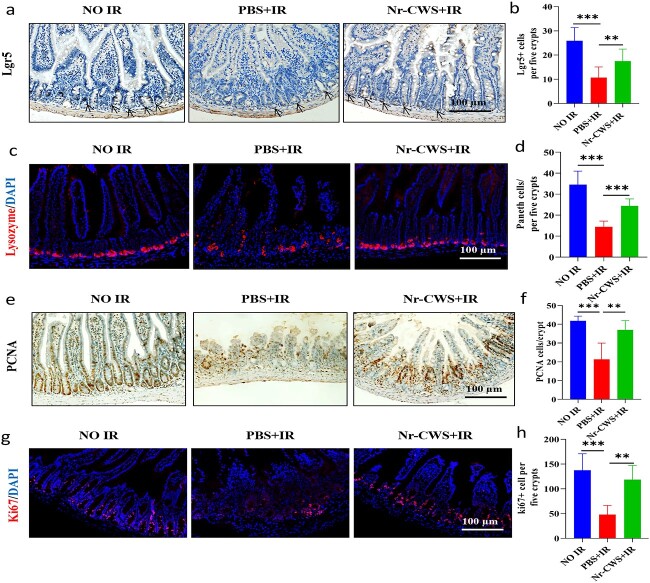
Nr-CWS enhances Lgr5+ ISCs survival and maintains the regeneration of intestinal epithelial cells in irradiated mice. Representative images of (**a**) Lgr5+ cells, black arrows, (**c**) lysozyme+, (**e**) PCNA+ and (**g**) Ki67 + (red, Ki-67+ and lysozyme+; blue, DAPI) in cross-sections of the intestine visualized by immunohistochemistry or immunofluorescence staining at 3.5 d after 12 Gy WAI. Scale bar: 100 μm. (**b** and **d**) Quantification of lgr5+ and lysozyme+ cells per five crypts, n = 8. (**f**) Quantification of PCNA+ cells per crypt, n = 3. (**h**) Quantification of Ki67+ cells per five crypts, n = 3. (^**^p < 0.01, ^***^p < 0.001) *Nr-CWS* nocardia rubra cell wall skeleton, *PBS* phosphate-buffered saline, IR ionizing radiation, *ISC* intestinal stem cell, *Lgr5* leucine-rich repeat-containing G protein-coupled receptor 5, *PCNA* proliferating cell nuclear antigen, *DAPI* 4,6-diamidino-2-phenylindole

The intestine is the fastest-renewed organ in the body and the intestinal epithelium constantly self-renews to maintain homeostasis and can regenerate quickly after injury. Lgr5^+^ and Bmi1 have been independently identified to mark long-lived pluripotent intestinal stem cells (ISCs) by lineage tracing in mice [37]. Lgr5^+^, also known as Gpr49, marks mitotically active ISCs that exhibit exquisite sensitivity to canonical Wnt modulation, contribute to homeostatic regeneration and are quantitatively ablated by irradiation [38]. Therefore, we assessed the effects of Nr-CWS on the proliferation and differentiation ability of intestinal crypt stem cells via immunohistochemistry staining. The number of Lgr5^+^ cells decreased post-irradiation compared with the NO IR group. However, the number of Lgr5^+^ cells in the Nr-CWS + IR group was significantly higher than that in the PBS + IR group at 3.5 d post-radiation (Figure 5a, b). Paneth cells, as intestinal stem cell bodyguards within the crypt, are a specialized cell type that nurture and protect ISCs, and produce a wide variety of antimicrobial products, such as lysozyme, to maintain intestinal homeostasis [38,39]. Therefore, we evaluated lysozyme^+^ cells after 12 Gy WAI using IF staining and found a significant reduction in lysozyme^+^ cells in the intestinal crypts at 3.5 d after radiation. However, these changes were significantly reversed after Nr-CWS treatment (Figure 5c, d).

In addition to continuous self-renewal, the intestinal epithelium can regenerate upon damage induced by radiation or acute inflammation. To explore the potential effect of Nr-CWS on regeneration, we quantified the intestinal regeneration crypts by immunohistochemistry staining of PCNA (a proliferation marker). The results showed that the number of PCNA^+^ cells in the Nr-CWS + IR group was ~1.7 times that of the PBS + IR group in irradiated mice (Figure 5e, f). Crypt base columnar cells are usually positive for the proliferation marker Ki67. IF staining of Ki67^+^ recognizes proliferating cells, indicating that cells are in a circulating state and regenerating [31]. In Nr-CWS-treated mice, the expression of Ki67^+^ cells increased compared with that in the PBS + IR group (Figure 5g, h), indicating that the regenerative ability of intestinal epithelial cells recovered after IR-induced damage. Therefore, Nr-CWS might enhance Lgr5^+^ ISC survival and maintain the regeneration of intestinal epithelial cells in irradiated mice.

### Nr-CWS improves the abundance and constitution of intestinal bacterial flora in WAI mice

**Figure 6:**
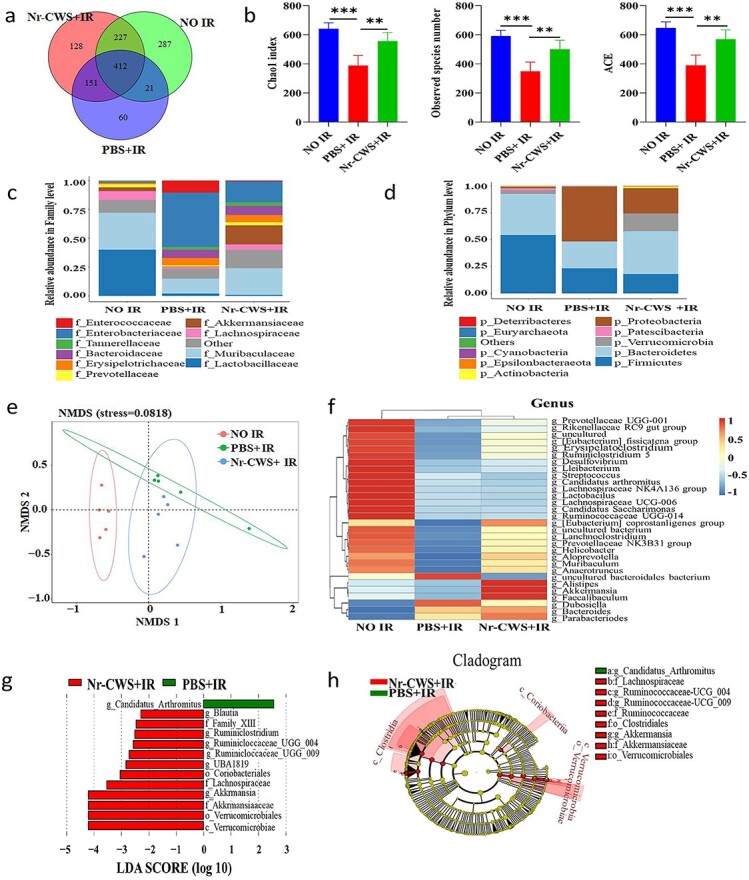
Nr-CWS treatment improves the abundance and constitution of gut microbiota in WAI mice. Feces were collected from the NO IR group mice and the irradiated mice treated with Nr-CWS/PBS at 3.5 d after 12 Gy WAI, and assessed by 16S rDNA high-throughput sequencing. (**a**) Venn diagram displaying the overlaps among groups. (**b**) Chao1 index, the observed species number and ACE of intestinal bacterial flora. (**c**) Relative abundance of the top 10 gut microbiota at the family level in mice. (**d**) Relative abundance of the top 10 gut microbiota at the phylum level in mice. (**e**) Cluster analysis by using non-metric multi-dimensional scaling (NMDS). Each spot represents one sample, the distance between points represents the degree of difference, and each group of mice is labeled by a different color. (**f**) Heat map analysis of abundance clustering at genus level; the heat map shows the top 30 genera ranked on the basis of abundance. Each column in the heatmap represents one group and each row represents one genus. The color bar showing blue to red indicates the relative abundance of each genus. (**g**) Linear discriminant analysis (LDA) effect size (LEfSe) results show that bacterial abundances were obviously different between the PBS + IR and Nr-CWS + IR groups, and indicate the effect size of the most differentially abundant bacterial taxon in the intestine. The length of the histogram represents the size of the LDA value, and the threshold on the logarithmic LDA score for discriminative features was 2. (**h**) Cladogram derived from LEfSe analysis of 16S rDNA sequences. Regions in green indicate clades that were enriched in PBS + IR group, while regions in red indicate clades that were enriched in Nr-CWS + IR group. Regions in yellow indicate clades with no significant difference. From inside to outside, the cladogram denotes the taxonomic level of phylum, class, order, family and genus. n = 5 (^**^p < 0.01, ^***^p < 0.001). *Nr-CWS* nocardia rubra cell wall skeleton, *PBS* phosphate-buffered saline, *IR* ionizing radiation, *OTU* operational taxonomic unit, *LDA* linear discriminant analysis

Mounting evidence has suggested a potential correlation between gut microbiota and IR-induced damage [40]. To verify the conjecture that Nr-CWS may play a role in resisting IR-induced injury by regulating intestinal flora, we used 16S rDNA gene sequencing to explore the variation in gut microbiota in irradiated mice with or without Nr-CWS treatment. First, we analyzed the common or unique OTUs in each group through OTU clustering results; the Venn diagram (Figure 6a) showed that there were 287, 60 and 128 unique OTUs in the NO IR, PBS + IR and Nr-CWS + IR groups, respectively, and the three groups contained 412 common OTUs. Moreover, we found that radiation caused obvious dysbiosis of the gut microbiota at 3.5 d post-WAI with a notable decrease in *α* diversity. The Chao1 index, observed species number and Abundance-based coverage estimator (ACE) reflect that the effect of α diversity was markedly reduced in the PBS + IR group, while these indices were restored in irradiated-mice treated with Nr-CWS (Figure 6b). Furthermore, IR also altered the composition of the intestinal flora at the family and phylum levels (Figure 6c, d). At the family level, we found that IR decreased the relative abundance of Lachnospiraceae, Akkermansiaceae and Prevotellaceae, whereas treatment with Nr-CWS restored that abundance. At the phylum level, the microbial community was dominated by Firmicutes, Bacteroidetes and Proteobacteria; IR increased the relative abundance of Proteobacteria and decreased that of Bacteroidetes, while treatment with Nr-CWS reversed this trend. Principal coordinates analysis by the NMDS method reflected that the structures of intestinal bacteria were changed after IR exposure and Nr-CWS treatment (Figure 6e). In Figure 6f we found that 12 Gy WAI exposure elevated the relative abundance of uncultured Bacteroidetes bacterium and Dubosiella at the genus level; however, treatment with Nr-CWS restored the abundances to the approximate levels of the control cohort, and Nr-CWS treatment improved the relative abundance of Alistipes, Akkermansia and Faecalibaculum, which have been shown to effectively alleviate intestinal inflammation [41,42]. To compare the intestinal bacterial flora, LEfSe calculations indicated the differences in taxa abundance at the genus level between the PBS + IR and Nr-CWS + IR groups (Figure 6g, h). We found that Candidatus_Arthromitus was obviously more abundant in the PBS + IR group than in the Nr-CWS + IR group, whereas Verrucomicrobiae, Akkermansiaceae and so on were enriched in the Nr-CWS + IR group. Hence, Nr-CWS treatment affected the abundance and composition of the intestinal bacterial flora, and the Nr-CWS-induced radioprotective effect might be mediated by some specific subset of bacterial taxa.

### Nr-CWS protects against radiation-induced spleen injury

**Figure 7:**
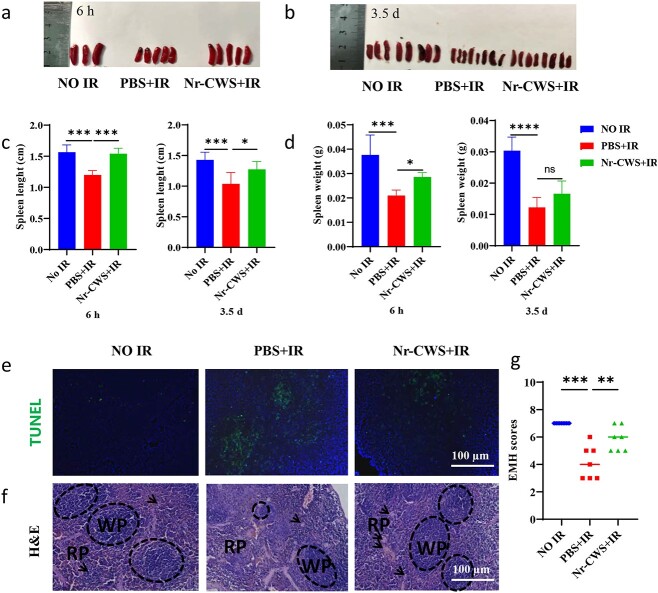
Treatment with Nr-CWS protects against radiation-induced spleen injury. (**a**, **b**) Spleen anatomy of mice at 6 h and 3.5 d after 12 Gy irradiation. (**c**) Length of spleens, n = 3–8. (**d**) Ratio of weight of dissected spleens, n = 3–8. (**e**) Apoptosis of the spleen was evaluated by a TUNEL assay of 6 h post-12 Gy WAI. Scale bar: 100 μm, n = 3/5/5. (**f**, **g**) Spleens 3.5 d post-radiation were stained with H&E and quantified for spleen extramedullary hematopoiesis scores. WP (white pulp), black dashed circles; RP (red pulp), area outside of WP, megakaryocytes, black arrows. Scale bar: 100 μm. n = 7. (ns, non-significance, ^*^p < 0.05, ^**^p < 0.01, ^***^p < 0.001). *Nr-CWS* nocardia rubra cell wall skeleton, *PBS* phosphate-buffered saline, *IR* ionizing radiation, *EMH* extramedullary hematopoiesis, *H&E* hematoxylin and eosin

In recent years, studies have indicated that Nr-CWS can regulate the body’s immune response and play an active role in treating a variety of malignant tumors [18,35]. The spleen is the body’s largest lymphoid organ, whose primary immunological function is to filter blood by intercepting microorganisms in blood via an immune response. Therefore, we next explored the effects of Nr-CWS on the spleen and EMH in irradiated mice. By observing the anatomical diagram of spleen organ separation, it can be seen that irradiated mice can reduce spleen atrophy after Nr-CWS treatment (Figure 7a, b). In addition, we found that the spleen length and spleen index of irradiated mice at 6 h and 3.5 d became shorter and decreased compared with those of the NO IR group, but treatment with Nr-CWS reversed this change (Figures 7c, d). Interestingly, pathological analyses revealed that IR damaged splenic white pulp (WP) and red pulp (RP). In the Nr-CWS + IR group, the splenic architecture was near typical normal, with WP containing well-developed and RP displaying extramedullary hematopoiesis at 3.5 d in irradiated mice (Figure 7f, g). Then, TUNEL staining of the spleen showed that irradiation caused cell apoptosis mainly in WP, while Nr-CWS treatment significantly reduced the number of apoptotic cells at 6 h post-radiation (Figure 7e). These results implied that Nr-CWS also protected the morphology and function of the spleen tissue from radiation-induced spleen damage.

### Nr-CWS-induced radioprotection is macrophage-dependent in irradiated mice

**Figure 8:**
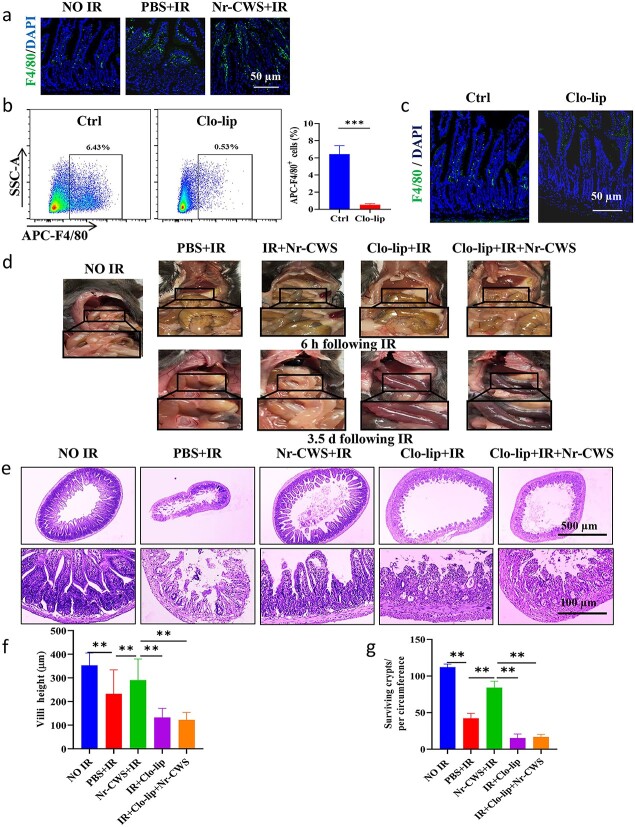
Nr-CWS-induced radioprotection is macrophage-dependent in mice. (**a**) Representative images of F4/80+ immunofluorescence staining in intestine sections at 3.5 d after 12 Gy WAI. Green, F4/80+; blue, DAPI. Scale bar: 50 μm. (**b**) Representative flow cytometry image of APC-F4/80 cells in spleen at 3 d after PBS liposome or clodronate liposome injection,in which the proportion of APC-F4/80+ positive cells was in the small square, and quantitative analysis the proportion of APC-F4/80+ in mice treated with PBS liposome and clodronate liposome, n = 3. (**c**) PBS liposome-treated and clodronate liposome-treated mice for 3 d. Representative images of F4/80+ immunofluorescence staining in intestine sections. Scale bar: 50 μm. (**d**) Gross images of intestinal anatomy at different times 6 h and 3.5 d post-radiation. (**e**) H&E staining of mice intestine sections after 12 Gy WAI. Scale bars: 500 μm (top) and 100 μm (bottom), n = 4. (**f**) Villus height at 3.5 d after 12 Gy WAI. (**g**) Average number of surviving crypts per intestinal circumference at 3.5 d after 12Gy WAI. (^**^p < 0.01, ^***^p < 0.001). *Nr-CWS* nocardia rubra cell wall skeleton, *PBS* phosphate-buffered saline, *IR* ionizing radiation, *Ctrl* control, *Clo-lip* clodronate liposome, *DAPI* 4,6-diamidino-2-phenylindole, *H&E* hematoxylin and eosin

The immune system plays a significant role in the healing process of tissue damage. Macrophages are immune cells that regulate the immune response and microbial homeostasis. Studies have reported that Nr-CWS can promote the activity of macrophages [43,44] and may have an effect on the host immune system [45]. Therefore, we investigated the effect of Nr-CWS on macrophages. F4/80 IF staining (Figure 8a) showed that the number of F4/80-positive cells in the intestine of irradiated mice increased significantly after Nr-CWS treatment. We further evaluated whether Nr-CWS-induced radioprotection required the involvement of macrophages. First, we treated mice with clodronate liposomes (Figure 8b), which effectively depleted macrophages in the body. To verify whether macrophages in the intestine were also depleted, F4/80 staining of intestinal sections showed (Figure 8c) that clodronate liposomes also depleted macrophages in the intestine. The model of radiation-induced intestinal injury was established by using macrophage-depleted mice exposed to 12 Gy WAI. Our results (Figure 8d) showed that the contents of the intestine were reduced and bloody diarrhea appeared in irradiated mice; the phenomena were more serious at 3.5 d compared with 6 h after radiation exposure. Compared to clodronate liposomes alone and clodronate liposomes combined with Nr-CWS treatment, Nr-CWS treatment did not improve radiation-induced intestinal content reduction and bloody diarrhea. These phenomena were confirmed by H&E staining (Figure 8e). Compared to irradiated mice receiving clodronate liposomes alone, the intestinal morphology of mice receiving clodronate liposomes followed by Nr-CWS treatment was also destroyed, and the villus height (Figure 8f), surviving crypts (Figure 8g) and epithelial integrity were not improved. These data suggest that Nr-CWS-mediated mitigation of radiation-induced intestinal damage is macrophage dependent.

### Nr-CWS activates the NF-κB signaling pathway and promotes the phagocytosis and migration ability of macrophages

**Figure 9:**
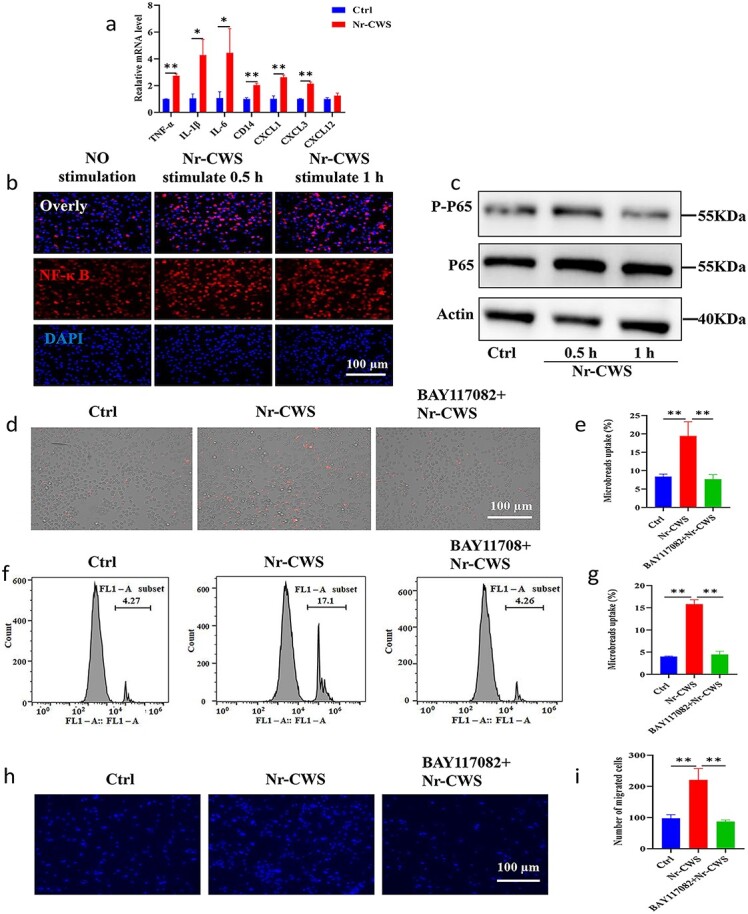
Nr-CWS stimulates macrophages and regulates their function. (**a**) mRNA levels of secretory factors and chemokines from PMs after treatment with 0.1 μg/ml Nr-CWS for 24 h. (**b**) PMs were treated with 0.1 μg/ml for 0.5 h and 1 h, incubated with NF-κB (P65) antibody overnight and the fluorescent secondary antibody for 1 h and then measured using fluorescence microscopy. Scale bar: 100 μm. (**c**) Isolated PMs were treated with 0.1 μg/ml Nr-CWS for different time, the protein expression levels of PP65 and P65 in PMs were detected by Western blot. (**d**) Representative images of PMs pretreated with 10 μM BAY-117082 for 2 h and treated with 0.1 μg/mL Nr-CWS for 24 h, and then incubated with latex microbeads for another 0.5 h measured by fluorescence microscopy. Scale bar: 100 μm. (**e**) Uptake analysis based on the images was measured using fluorescence microscopy, n = 3. (**f**) Representative flow cytometry images of PMs pretreated with 10 μM BAY-117082 for 2 h and treated with Nr-CWS for 24 h and then incubated with latex microbeads for another 0.5 h. (**g**) Uptake of latex microbeads was measured using flow cytometry, n = 3. (**h**) PMs were pretreated with 10 μM BAY-117082 for 2 h and with 0.1 μg/ml Nr-CWS for 24 h in transwell plates, and incubated with crystal violet for ~20 min. Representative images were measured using fluorescence microscopy. Scale bar: 100 μm. (**i**) Quantification of the number of migrating macrophages, n = 3 plates of PMs per group and random observation of three visual fields per plate. (^*^p < 0.05, ^**^p < 0.01). *PBS* phosphate-buffered saline, *IR* ionizing radiation, *Ctrl* control, *P65* nuclear factor kappa-B, *PP65* phosphoprotein nuclear factor kappa-B

*In vivo* studies have shown that the radioprotective effect of Nr-CWS depends on macrophages. To further clarify the potential modulatory effect of Nr-CWS on macrophages, we found that Nr-CWS increased the mRNA levels of Tumor necrosis factor alpha (TNF-α), interleukin (IL)-1β, IL-6, CD14, CXCL1 and CXCL3 *in vitro* (Figure 9). NF-κB plays a vital role in the cellular and organismal response to infectious agents as a mediator of innate and adaptive immune responses [46]. Chou *et al*. [47] demonstrated that activation of NF-κB selectively protects the intestine from radiation-induced damage. PMs treated with Nr-CWS for 24 h were detected by IF staining, which showed the migration of NF-κB to the nucleus after treatment with Nr-CWS for 0.5 h; however less migration of NF-κB was found in the control group (Figure 9b). In addition, phosphorylation of NF-κB occurred (Figure 9c) when PMs were treated with Nr-CWS. Given the critical role of macrophages in bacterial clearance, we speculated on the effect of Nr-CWS on macrophage phagocytosis by monitoring the uptake of latex microbeads. PMs treated with Nr-CWS for 24 h were observed to have an apparent increase in the uptake of latex microbeads (Figure 9d, e), which was also supported by the flow cytometry results (Figure 9f, g). Moreover, the effect of Nr-CWS on macrophage migration was observed, and the results showed (Figure 9h, i) that Nr-CWS could significantly promote the migratory ability of macrophages. To verify whether Nr-CWS-promoted macrophage phagocytosis and migration was dependent on NF-κB activation, we pretreated PMs with the NF-κB inhibitor BAY-117082 for 2 h and then treated them with Nr-CWS. We found that BAY117082 significantly inhibited the promoting effect of Nr-CWS on macrophage phagocytosis and migration (Figure 9d–i). The above results indicated that Nr-CWS could activate the NF-κB signaling pathway and promote the phagocytosis and migration ability of macrophages.

## Discussion

Radiation is a nonspecific and widely applied therapeutic method for malignant tumors. Patients with colorectal cancer and other abdominal tumors have received radiation therapy, and ~60–80% of these patients suffer from radiation-induced intestinal injury [46]. Many reports have suggested that hematopoietic stem cell transplantation or growth factor administration is an effective treatment for patients with bone marrow radiation sickness [47]. However, there is no effective treatment strategy for radiation-induced intestinal injury. Therefore, developing effective therapies and drugs to prevent or treat irradiation-induced gastrointestinal toxicity is urgently needed, and scholars have carried out various explorations and screenings on radiation-induced intestinal injury.

With the in-depth study and exploration of radiation damage and protection strategies, immune regulation has attracted wide attention and has been proven to be an effective strategy [1,18,48]. For example, evidence confirmed that lyceum babayum played a key protective role in radioprotection by regulating the immune response and gut microdiota and related metabolites [49]. Another study indicated that naringenin could serve as an immunomodulator to ameliorate radiation-induced lung injury by lowering IL-1 [48]. It has been reported that Nr-CWS is not only an immunomodulator but also an anti-tumor agent [27], consisting of arabinogalactan, mycolic acid and mucopeptide and enhancing the ability of the host to clear infection. Studies have demonstrated that Nr-CWS has antitumor [14], immune response regulation [23,29,50], angiogenesis and cutaneous wound healing promoting properties [51]. A study proved that treatment with Nr-CWS could reduce Programmed cell death 1 ligand 1 (PD-L1) in tumor tissues and provide the possibility for the combination of Nr-CWS with PD-1/PD-L1 antibodies [43]. Therefore, we speculated that Nr-CWS might have potential value in resisting the side effects of ionizing radiation exposure.

To evaluate the above hypothesis, we first confirmed whether Nr-CWS might decrease IR-induced intestinal injury. IR induces substantial DNA damage and cell apoptosis, which lead to gastrointestinal and hematopoietic damage [4]. Previous reports have shown that WAI damages not only the gastrointestinal system but also other systems. The mechanism may be related to the soluble factors secreted by radiation cells [52]. Moreover, another mechanism may be related to the radiation-induced bystander effect [53]. For example, Parsons *et al*. [54] reported damage to the bone marrow in children’s sternum while receiving IR to the spleen for chronic granulocytic leukemia. In the present study, we also found that WAI damaged not only the intestine but also spleen tissue. Our results demonstrated that Nr-CWS treatment improved the survival of mice following 12 Gy WAI. Nr-CWS treatment kept the intestinal crypt villus well preserved and decreased DNA damage and cell apoptosis in the irradiated mice. Under normal physiological conditions, Lgr5^+^ ISCs located at the bottom of the crypt supplement intestinal epithelial cells and are shed from villus tips an average of 5 d after ‘birth’ [55,56]. A study indicated that Lgr5^+^ ISCs are indispensable for intestinal regeneration following radiation exposure [57]. In contrast to other intestinal epithelial cell types, Paneth cells move downward after differentiation. They can be found exclusively at the bottom of the intestinal crypt and are maintained for up to 2 months [53]. Paneth cells act as stem cell bodyguards within the crypt, a specialized cell type that nurture and protect ISCs [55]. We found that treatment with Nr-CWS promoted the Lgr5^+^ cell levels and maintained the regenerative capacity of intestinal epithelial cells, including lysozyme+ Paneth cells, PCNA^+^ cells and Ki67^+^ transient amplifying cells. Hence, we concluded that Nr-CWS treatment alleviated IR-induced intestinal damage, maintained the structure and function of crypt villi, enhanced Lgr5^+^ ISC survival and improved the regeneration of intestinal epithelial cells.

Healthy gut microbiota is significantly important for immune regulation and nutrient absorption [58]. Presently, some articles have indicated a potential relationship between intestinal flora dysbiosis and IR-induced injury. Intestinal microbial dysbiosis affects the host’s ability to resist ionizing radiation [59,60]. A study proved that the ‘elite-survivors’ of irradiated mice harbored distinct gut microbiota that developed after radiation and protected against radiation-induced damage and death [31]. The abundances of the intestinal bacterial taxa Lachnospiraceae and Enterococcaceae were associated with post-radiation recovery of hematopoiesis and gastrointestinal repair [31,61]. We determined the abundance and composition of irradiated mice by 16S rDNA gene sequencing. We found that IR obviously decreased the abundance and composition of the intestinal bacterial flora, while treatment with Nr-CWS changed and improved the abundances of beneficial bacterial taxa, such as Lachnospiraceae, Akkermansia and Bacteroideles. Notably, the effects of the intestinal flora shaped by Nr-CWS on radioprotection are complicated, which may be due to basic intestinal conditions, body immune status and other factors.

Intestinal macrophages have complex functions, high heterogeneity and plasticity, and play a key role in the immune response, contributing to the initiation and regression of inflammation and the coordination of tissue repair [62]. In intestinal homeostasis, macrophages remove pathogenic bacteria and apoptotic or senescent cells and maintain intestinal immune homeostasis [63]. In addition, intestinal macrophages can produce a variety of cytokines and soluble mediators, which are involved in the physiological activity and repair of intestinal epithelial cells, intestinal stem cells and intestinal neurons [64]. Increased numbers of macrophages were observed several days after irradiation exposure. It has been proposed that macrophage activation is a secondary effect of IR, which results from cellular damage signals and the clearance of radiation-induced apoptotic cells, rather than a direct effect of IR [65]. In recent years, macrophage-based therapeutic interventions against radiation damage have gained significant attention. A study demonstrated that a rutin formulation (G-003 M) can modulate the proinflammatory programming of macrophages and mitigate radiation-induced inflammatory stress to resist radiation damage [66]. Another study indicated that Lactobacillus rhamnosus GG (LGG) protects the intestinal epithelium from radiation injury through the release of lipoteichoic acid, macrophage activation and the migration of mesenchymal stem cells, and depletion of macrophages blocked LGG-induced mesenchymal stem cell migration and radioprotection [67]. Some studies have shown that macrophages interact with the intestinal flora. Macrophages can be persistently activated by sustained exposure to microbes and their metabolites [68–70]. Intestinal macrophage depletion can lead to decreased macrophage phagocytosis and defense responses [71]. In addition, intestinal macrophages also play a crucial role in shaping the gut microbiota. A study showed that macrophage deficiencies may prevent the establishment of the core microbiota, thereby allowing the outgrowth of rare and unusual bacteria [72]. In our study we found that Nr-CWS-induced radioprotection promoted recovery of the intestinal flora in irradiated mice in a macrophage-dependent manner. Macrophage depletion blocked Nr-CWS-induced intestinal protection. These results offer novel insight into the treatment of radiation-induced intestinal injury.

NF-κB, an important nuclear transcriptional regulation factor, participates in the transcription regulation of diverse genes and actively contributes to the immune response and cell cycle activity. NF-κB not only plays a key role in the inflammatory response but also plays a protective role in intestinal epithelial cell injury. Activation of NF-κB can induce multiple factors that contribute to cell protection and promotetissue regeneration, including reactive oxygen species scavengers, apoptosis inhibitors, cellsurvival and cytokines [73]. Burdelya *et al*. [74] studied CBLB502, a polypeptide drug derived from Salmonella flagellin that binds to toll-like receptor 5 and activates NF-κB signaling, and suggested it as a biologic protectant in radiation emergencies. Interestingly, in this study, we found that Nr-CWS treatment activated NF-κB signaling and promoted the phagocytosis and migration ability of macrophages *in vitro*. NF-κB inhibitors effectively inhibited the effect of Nr-CWS on macrophage function. However, the mechanisms underlying intestinal protection against IR by Nr-CWS need further research.

Collectively, our results show that Nr-CWS, as an immunopotentiator, was used to treat radiation-induced intestinal injury in mice. Interestingly, we found that Nr-CWS had a significant radio-protective effect against radiation that was macrophage dependent. Nr-CWS could activate the NF-κB signaling pathway and promote the phagocytosis and migration function of macrophages.

## Conclusions

In summary, our findings demonstrated that Nr-CWS possesses potential properties against radiation-induced acute intestinal injury. Nr-CWS, as an approved National Category II New Drug, might be a promising therapeutic candidate drug for patients undergoing accidental exposure or radiotherapy.

**Abbreviations**
AB-PAS: Aligen blue Periodic-acid-Schiff; DAPI: 4,6-diamidino-2-phenylindole; EMH: extramedullary hematopoiesis; H&E: Hematoxylin and eosin; γH2Ax: phosphorylated histone 2Ax; IF: Immunofluorescence; i.p.: Intraperitoneally; IR: Ionizing radiation; ISCs: Intestinal stem cells; LDA: Linear discriminant analysis; LEfSe: Linear discriminant analysis effect size; NF-κB: Nuclear factor kappa-B; NMDS: Nonmetric multidimensional scaling; Nr-CWS: Nocardia rudra cell well skeleton; OUT: Operational taxonomic unit; PBS: Phosphate-buffered saline; PCNA: Proliferating cell nuclear antigen; PM: Peritoneal macrophage; RP: Red pulp; TUNEL: TdT-mediated dUTP nick-end labeling; WAI: Whole abdominal radiation; WP: White pulp; ZO-1: Zonula occludins-1.

## Funding

This work was supported by the Key Program and Major Program of the National Natural Science Foundation of China (No. 82030056 and 82192884).

## Data availability

Data are available from the authors upon reasonable request.

## Authors’ contributions

CS, LW and Yu Wang designed, carried out and analyzed the data and wrote the manuscript with input from all coauthors. LC, HL and KX under took the immunohistochemistry/immunofluorescence experiments. CS conceived and supervised the study. YW, WC and AZ analyzed and interpreted data from experiments. All authors discussed the results and commented on the manuscript.

## Ethics approval and consent to participate

This study of all experiments involving animals were approved by the Ethics Committee and performed in accordance with the Animal Care and Use Committee Guidelines of the Army Military Medical University (Chongqing, China).

## Conflicts of interest

None declared.

## Supplementary Material

supplementary_data_tkad045
