# Supplementary material for: Nocardia rubra cell-wall skeleton mitigates whole abdominal irradiation-induced intestinal injury via regulating macrophage function
Source: Burns Trauma. 2024 Mar 4;12:tkad045. doi: 10.1093/burnst/tkad045 (PMC10914217; doi:10.1093/burnst/tkad045)

**Spleen scoring system was based on the amount and patterns of extramedullary hematopoiesis (EMH) as described here:**

1.Atrophy;

2.Atrophic white pulp with less than 20%;

3.Atrophic white pulp with 20-60%;

4.Atrophic white pulp with 60-100%

5.Early white pulp recovery with extensive EMH in red pulp;

6.Well developed white pulp with extensive EMH in red pulp;

7.Normal spleen.


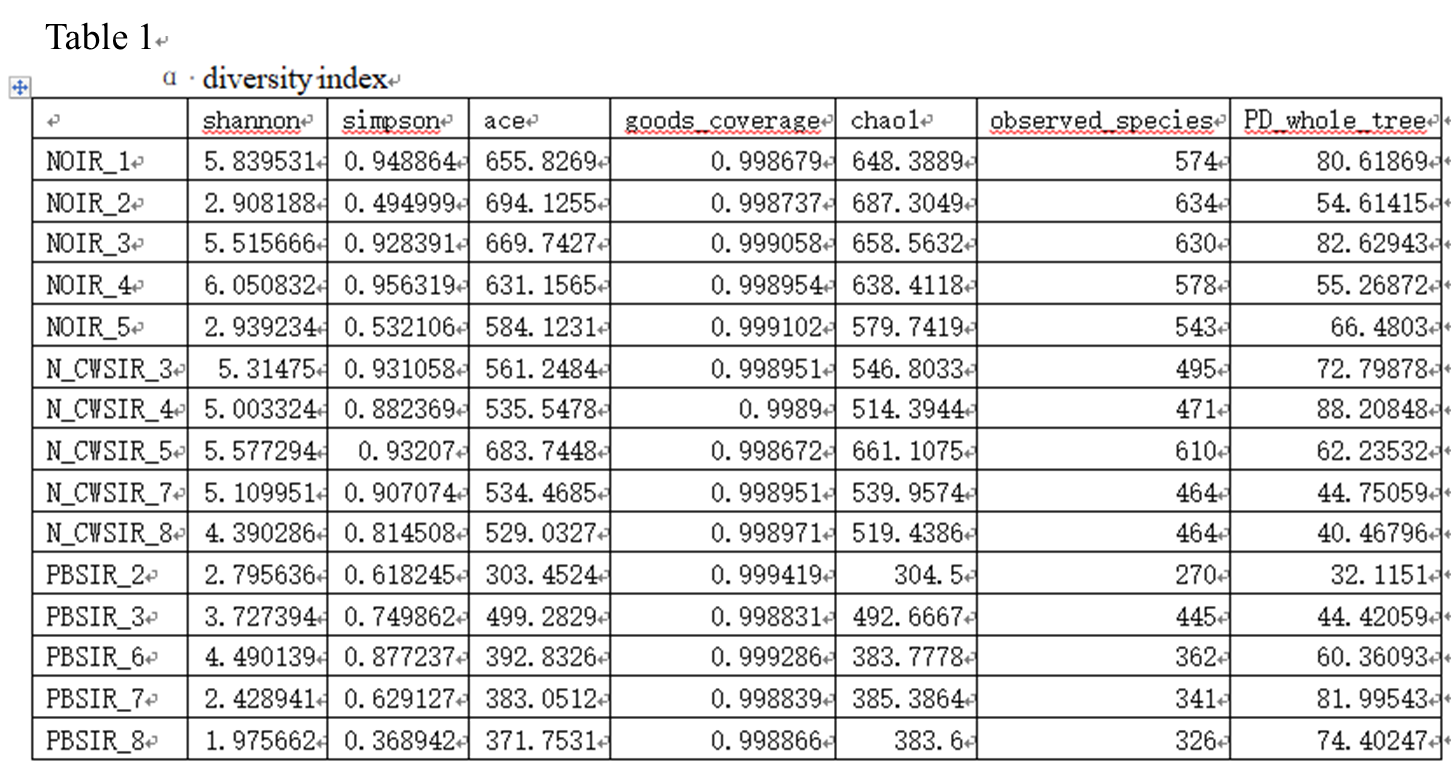


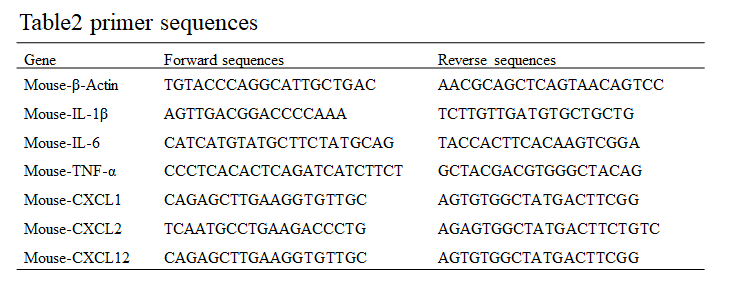

Supplement: supplementary_data_tkad045 [file supplementary_data_tkad045.docx]
